# Problematic internet usage: the impact of objectively Recorded and categorized usage time, emotional intelligence components and subjective happiness about usage

**DOI:** 10.1016/j.heliyon.2022.e11055

**Published:** 2022-10-13

**Authors:** Sameha Alshakhsi, Khansa Chemnad, Mohamed Basel Almourad, Majid Altuwairiqi, John McAlaney, Raian Ali

**Affiliations:** a College of Science and Engineering, Hamad Bin Khalifa University, Qatar; b College of Technological Innovation, Zayed University, United Arab Emirates; c College of Computers and Information Technology, Taif University, Saudi Arabia; d Faculty of Science and Technology, Bournemouth University, UK

**Keywords:** Problematic interenet usage, Smartphone apps usage, Trait emotional intelligence, Internet addiction, Addictive behavior, Digital wellbeing

## Abstract

Most research on Problematic Internet Usage (PIU) relied on self-report data when measuring the time spent on the internet. Self-reporting of use, typically done through a survey, showed discrepancies from the actual amount of use. Studies exploring the association between trait emotional intelligence (EI) components and the subjective feeling on technology usage and PIU are also limited. The current cross-sectional study aims to examine whether the objectively recorded technology usage, taking smartphone usage as a representative, components of trait EI (sociability, emotionality, well-being, self-control), and happiness with phone use can predict PIU and its components (obsession, neglect, and control disorder). A total of 268 participants (Female: 61.6%) reported their demographic and completed a questionnaire that included Problematic Internet Usage Questionnaire short form (PIUQ–SF–6), Trait Emotional Intelligence Questionnaire-Short Form (TEIQue-SF), level of happiness with the amount and frequency of smartphone use, and living conditions (whether alone or with others). Their smartphone usage was objectively recorded through a dedicated app. A series of one-way ANOVA revealed no significant difference in PIU for different living conditions and a significant difference in the subjective level of happiness with phone usage (F (3, 264) = 7.55, p < .001), as well as of the frequency of usage where the unhappy group had higher PIU (F (3, 264) = 6.85, p < .001). Multiple linear regression analysis showed that happiness with phone usage (β = −.17), the actual usage of communication (β = .17), social media (β = .19) and gaming apps (β = .13), and trait EI component of self-control (β = −.28) were all significant predictors of PIU. Moreover, gender, age, and happiness with the frequency of phone usage were not significant predictors of PIU. The whole model accounted for the total variance of PIU by 32.5% (Adjusted R^2^ = .287). Our study contributes to the literature by being among the few to rely on objectively recorded smartphone usage data and utilizing components of trait EI as predictors.

## 1 Introduction

Technology in its different forms has become a core pillar in almost every aspect of our lives, from work, school, and social interactions, to entertainment, health, education, and much more. From 2000 to 2021, internet use increased by 1,331.9%. In early 2020, global internet traffic increased from 20% to 70% [1], and 92.6% of internet users accessed the internet using their smartphones [2]. Although using the internet benefits people, such as by enabling them to work from home and to socialize as an alternative to meeting in person, its excessive use is problematic to daily functioning [3, 4] and can negatively impact their physical and mental health. Such an effect is more notable when the internet is used for leisure, leading to conflicts with people's life responsibilities and priorities [5, 6].

Internet overuse and its associated psychological problems were proposed in 1995 as an Internet Addiction Disorder [7]. Although internet addiction has not been formally diagnosed as a disease, the research argued that it could be considered an addictive behavior [8]. Internet addiction has no standardized definition or framework, with various terms being used in the literature, including Pathological Internet Use, Compulsive Internet Use, and Problematic Internet Use (PIU) [9]. PIU, the term used in this study, is defined in literature as excessive internet use that negatively impacts a person's life in one or more of its important facets [10]. Over the past years, studies have reported the negative consequences of PIU [11]. In addition to the negative impact on mental health, studies found significant associations between PIU and negative academic performance [12], physical problems [13], and reduced life satisfaction [14]. Previous studies also found an association between problematic use of a certain app type with one's well-being and poor sleep quality [15, 16].

Extensive research has studied the factors associated with PIU and the process of its development, which shall help devise measures that can reduce its impact and improve people's lives. For example, a study conducted at a Canadian university revealed several parameters that can predict PIU, including a low level of well-being, gender, marital status, ethnic background, and heavy internet use for communication purposes [11]. Other studies revealed more parameters associated with PIU, such as social support, cognitive confidence, and emotion regulation [17], coping style [18], self-esteem [19], boredom [20], loneliness, and shyness [21, 22], lack of family love [23], peer influence [24], and daily internet use time [25]. The daily amount of time spent using the internet is one of the important indicators of PIU [26] and has been recommended as a diagnostic criterion for PIU [27]. However, most of the studies in the literature that investigated this relation between PIU and internet use time were dependent on self-reporting measurements, that is, participants stated the amount themselves, typically through a questionnaire. Self-reporting could lead to biased results because participants are more likely to overestimate or underestimate their amount of usage [28]. The present study addresses this limitation by collecting smartphone usage data objectively through a designated app.

Early theories compared PIU to substance use addiction and suggested the time spent on the internet as a diagnostic criterion for PIU [29, 30]. The literature examined the association of the amount of time spent on the internet, as reported by the participants, with PIU and revealed different inconsistent findings. While some studies found a significant and positive association between time spent on the internet and PIU [31, 32], findings in [33] revealed no significant difference. Previous studies showed that time spent on the internet is significantly associated with PIU when spent for non-essential purposes such as non-academic activities [34]. Concerns were raised in the late 1990s when studies started to argue that PIU is not about addiction to the internet but rather addiction on the internet [35, 36]. In other words, PIU should take into consideration the type of online activities the user spent time on (e.g., communication, social networking, gaming, shopping, etc.) and their reasons for doing so, such as escapism and fear of being excluded, rather than looking only at the total amount of time spent on the internet. Empirical studies focusing on studying the association between the type of internet usage and PIU started to emerge in the last decade, and their findings were still inconsistent. According to Kumar et al. [37], the social media usage amount reported by the study participants was dominant in predicting PIU, while the time spent on gaming did not predict PIU, which could be explained by the popularity and widespread use of social networking apps [36, 38] but not gaming which requires larger screens and dedicated devices, relatively more expensive. Another study showed that time spent on gaming for males and online streaming for females were predictors of PIU [39]. A study in [40] found associations between PIU and gaming usage at night only, denoting conflict with other life priorities such as sleep. Other studies found that times spent on entertainment, gaming, and communication were associated with PIU [41, 42]. The reliance on self-reported usage time in these studies, e.g., through a survey, is a major limitation. Self-reporting may entail a distorted perception of use by the user [43]. Hence, in our paper, we rely on objectively recorded usage time.

Empirical studies using objective data to investigate the relationship between internet use and PIU started to appear. The amount of studies that utilized this kind of approach to measure screen time is still limited [44, 45]. Recently, applications known as ‘digital wellbeing’ [46] have become available to track usage and make it available to users and potentially to researchers. Still, it remains technically challenging to get the data without dedicated application programming interfaces (APIs), especially for enabling real-time responses and interventions. Another reason for the scarcity of research that utilized objective data recording relates to people's concern about the privacy and anonymity of their internet usage [4]. Relying on objective monitoring of smartphone internet use enables the collection of more precise and valid data. The study in [47] compared self-reported and objectively measured time spent on smartphones and found that the self-reporting measures had low validity. However, studies that relied on objective data, besides being scarce, have limitations such as the small sample size, the focus on the student population, the use of apps developed specifically for tracking usage leading to influencing participants' usage behavior, and their exploration of the correlation between variables without applying regression or classification [40, 48]. Our work attempts to address these methodological issues and research gaps in the literature.

Emotional Intelligence (EI) has been defined as the ability to regulate emotions in one's self and others, employ feelings to plan, guide actions and achieve in life [49, 50]. On the other hand, trait EI has been described as emotional dispositions and self-perceptions [51]. EI has drawn much attention in psychology [52] as literature demonstrated that there is an association between EI and different factors such as mental health, well-being, social life, and work outcome [53]. EI was also found to have a relation with addictive behaviors, conceptualized as maladaptive behaviors that serve as a coping strategy to manage emotional difficulties [54]. According to the Interaction of Person-Affect-Cognition-Execution (I-PACE) model [55], considering that emotional difficulties are part of the core characteristics of a person, EI could interpret the development of PIU. Individuals with low EI are less likely to use adaptive strategies when faced with emotional difficulties and may navigate the internet to alleviate their emotions. An experimental study in [8] concluded that when participants experienced negative emotions, it increased their urge to access the internet, particularly social media. A few studies investigated the relationship between EI and PIU and found a significant negative relationship between PIU on the one hand and EI [56, 57] and its components [52] on the other hand. On these bases, the current study investigated the relation between EI components and PIU.

In this study, we investigate the relationship between the use of each type of applications, on the one hand, and PIU and its components, on the other hand. Participants' actual usage was collected objectively via an app that automatically collects smartphone usage and is already available on Google Play. Due to the ease of access to the internet through smartphones and the high percentage of internet users who access the internet via their smartphones, smartphone usage is considered a representative of technology usage, particularly non-essential usage, which is the main concern of PIU. It has also been shown that time spent on smartphones and time spent on the internet correlate [58]. We then aggregated the amount of smartphone usage and categorized it into communication apps usage, social media apps usage, gaming apps usage, and other apps usage. We then examined the impact of each component of EI (well-being, self-control, emotionality, and sociability) using the Trait Emotional Intelligence Questionnaire-Short Form (TEIQue-SF) scale [59] on PIU and its components (obsession, neglect, and control disorder) using the Problematic Internet Usage Questionnaire short form (PIUQ–SF–6) scale [60]. We considered other variables to characterize demographics, living conditions, and subjective feelings about technology use. The variables included gender, age (emerging adults and adults), living condition (alone or with others), and the user's level of happiness with the amount of phone use, as well as happiness with the frequency of checking the phone. Therefore, this paper aims to answer the following research questions:

(RQ1): Does PIU differ according to the different categories of age, gender, and living condition?
(RQ2): Can levels of subjective happiness with phone use, objectively measured amount of smartphone usage of each category of apps, and the four components of EI predict PIU?
(RQ3): Can levels of subjective happiness with phone use, objectively measured amount of smartphone usage of each category of apps, and the four components of EI predict the three components of PIU?

## 2 Materials and methods

### 2.1 Dataset

The data were collected objectively by monitoring the user's smartphone screen time and recording the usage via a dedicated smartphone application. The application explains in its privacy policy, to which all users agreed, its data collection process and that it shares data anonymously with academics and research partners. Nevertheless, during application installation, the users were explicitly asked if they agree to participate. The users who participated in the survey were offered a premium version of the application. A total of 602 users responded to the survey and agreed to take part in our study. Participants who had incomplete responses to the questionnaires of the current study or answered in quick time were excluded. Those who used the application for less than seven days and those for whom we had missing data in these first seven days were excluded. Only 268 participants (Female = 165) met the inclusion criteria. Our participants were from 10 countries (India, United States, United Kingdom, Canada, Australia, Germany, Netherlands, Brazil, France, and Sweden). Their ages ranged from 15 to 64, and the age question was answered using age range response options (15–24, 25–34, 35–44, 45–54, and 55–64). For this study, the ages were grouped into the emerging adults group, those in the age range of 15–24 as defined by the United Nations [61], and the adults group comprising those aged 25 and above. The sample data were collected between October 2020 to April 2021. This research study was approved by the Institutional Review Board (IRB) of Qatar Biomedical Research Institute (QBRI) at Hamad Bin Khalifa University (QBRI-IRB 2021-08-102).

### 2.2 Measures

#### 2.2.1 Demographics

The collected demographic information included gender, age, and living condition, specifically whether the participant is living alone, with a partner or family, with roommates, or prefers not to say. The data also captured the participant's level of happiness with phone use by using two items. The first item measured happiness with phone usage by asking the participants how they felt about the amount of time they used their phones per day. The second item measured happiness with the frequency of phone checking by asking the participants how they felt about the number of times they checked their phone per hour. The two items were on a four-point scale: 1: *Unhappy with it*, 2: *Somewhat unhappy with it*, 3: *Somewhat happy with it*, and 4: *Happy with it*.

#### 2.2.2 Problematic Internet Usage

PIU was measured via the Problematic Internet Use Questionnaire Short-Form (PIUQ–SF–6), which is a self-reporting scale proposed by Demetrovics et al. [60]. The scale consists of six items, making it a short form of the 18-item PIUQ scale [62]. The six items of the scale are listed in [63]. The responses are rated on a five-point Likert scale (from “*never*” to “*always/almost always*”). The scores of PIUQ–SF–6 ranged from 6 to 30, with higher values indicating increased PIU. The scale also assesses three components of PIU, namely, obsession, neglect, and control disorder, with each calculated based on two items of the six items. The obsession component represents the cognitive engagement with using the internet and the mental symptoms caused by the lack of use. The neglect component explains the extent to which an individual ignores their basic daily needs and activities due to their internet use. The control disorder component measures how difficult for a person to control their internet usage. The scale reliability was evaluated, and Cronbach's alpha value was 0.77 [60].

#### 2.2.3 Average of app types usage

The monitoring application employed in this study monitors and stores comprehensive details of the participant's smartphone usage while the phone screen is on, such as screen lock and unlock time, each session of using an application, application name, and session start and end timestamps. The participants' usage data for the first seven days were extracted, and the averages of the time spent on communication apps, social media apps, gaming apps, and other apps were calculated. The usage of a seven-day period was considered in this study as previous research concluded that a minimum of five days is required to reflect weekly usage behavior [64]. We also note that requiring more than seven days would have reduced our sample size because some participants started to uninstall the app after the first week. They were free to withdraw from the app and the study at any point.

#### 2.2.4 Emotional intelligence

EI was measured using Trait Emotional Intelligence Questionnaire short version (TEIQue-SF) [59], which was itself adapted from a long version (153 items) [65]; the questionnaire can be found on the author's website (https://psychometriclab.com/obtaining-the-teique/). TEIQue-SF consists of 30 questions that are used to measure the global EI trait score (from 1 to 7) along with four different components constituting emotional self-perception (components scores range from 1 to 7). *Emotionality* (8 items) measures the ability of an individual to perceive and connect with emotions in one's self and others. *Self-control* (6 items) measures the ability to regulate emotions, stress, and impulsive behavior. *Well-being* (6 items) measures personal feelings on achievements and expectations. *Sociability* component (6 items) measures whether the person perceives themselves as socially confident and effective in communication and participates in social events [66]. The reliability of TEIQue-SF has been established in the literature. The Cronbach's alpha was 0.89 for the global trait EI score and ranged from 0.67 to 0.92 for its component scores [67].

### 2.3 Data analysis

Millions of usage records were pre-processed using Python 3.8 [68]. The pre-processing procedure included removing duplicates, eliminating users with missing survey responses and demographic information, unifying date language and formats for all the sessions, merging sessions of the same application with 1 s or less time gap, and calculating the scales scores and overall usage average and that for the four app types. The data were analyzed using JASP version 0.16.0 [69]. PIU and each of its components, which are the dependent variables in the current study, were treated as a continuous variable, and normality was examined. The association between PIU and the living conditions, happiness with phone usage, and happiness with the frequency of phone checking variables were analyzed using a series of one-way ANOVAs followed by Tukey's post hoc test. A multiple regression analysis was performed to investigate the influence of gender, age, objectively recorded app types usage data, trait EI components, happiness with phone usage, and happiness with the frequency of phone checking in the prediction of PIU. Similarly, a series of multiple regression models was also performed to investigate the impact of the variables of interest in this study on the three components of PIU (obsession, neglect, and control disorder). Pearson's correlation analysis was also performed to examine the multicollinearity of variables.

## 3 Results

### 3.1 Sample characteristics

Descriptive statistics of the participants are shown in Table 1 and Table 2. Of 268 participants, 61.6% were female and 56.3% were adults. A high percentage of the participants (77.6%) lived with their family or a partner. The participants were from different professions: 47.8% were employed, 34.3% were students, and the rest did not mention their profession. Most of the participants reported being unhappy with the amount of time they spent using their phone (60.1%) and unhappy with the frequency of their phone checking (61.6%). The mean of PIU was 18.46 and of trait EI was 4.53. Participants’ daily average of smartphone usage was 303.05 min.

**Table 1:** Descriptive statistics of demographic.

Variables	N (268)	%
**Gender**		
Male	103	38.4
Female	165	61.6
**Age**		
Emerging Adults (15–24)	117	43.7
Adults (25–64)	151	56.3
**Living condition**		
By my own	30	11.2
With partner or family	208	77.6
With other roommate, e.g. student accommodation	26	9.7
Prefer not to say	4	1.5
**Happiness with the amount of time of using smartphone**		
Unhappy with it	161	60.1
Somewhat unhappy with it	64	23.9
Somewhat happy with it	34	12.7
Happy with it	9	3.4
**Happiness with the number of times of checking phone**		
Unhappy with it	165	61.6
Somewhat Unhappy with it	67	25.0
Somewhat Happy with it	26	9.7
Happy with it	10	3.7

**Table 2:** Descriptive statistics of smartphone usage and survey questionnaires N (268).

Variables	M	SD
PIU	18.46	4.63
Obsession	5.49	2.13
Neglect	6.09	1.84
Control Disorder	6.88	2.01
Avg smartphone usage (min)	303.05	157.30
Avg comm. usage (min)	44.21	41.26
Avg social media usage (min)	72.42	77.67
Avg gaming usage (min)	27.57	52.40
Avg other apps usage (min)	163.76	102.25
Global_EI	4.53	0.82
Well-being	5.03	1.14
Self-control	4.05	1.08
Emotionality	4.70	0.99
Sociability	4.39	1.05

### 3.2 Association between living status and PIU

The test assumptions for ANOVA were met for the living conditions variable. The one-way ANOVA result revealed no statistically significant difference on the PIU whether the participant lived alone, with a partner or family, or with a roommate (F (3, 264) = 1.20, and p = .311).

### 3.3 Association between happiness with smartphone usage and PIU

A one-way ANOVA test was also conducted to assess the differences of PIU across the different levels of users' happiness with time spent using the phone. The test assumptions for ANOVA were checked. Levene's test was not significant (p-value = .980), indicating no violation of the homogeneity of variance. Normality was checked with a Q-Q plot, where no deviations were noted. There was a significant difference among the four groups of happiness with the amount of phone usage (F (3, 264) = 7.55, p < .001, η^2^ =. 079). A follow-up post hoc analysis showed a significant difference between the Unhappy group (M = 19.46, SD = 4.41) and Somewhat Unhappy group (M = 17.42, SD = 4.70) with p = .012, the Unhappy group and Somewhat Happy group (M = 16.62, SD = 4.37) with p = .005, and the Unhappy group and Happy group (M = 15.00, SD = 4.18) with p = .020. The results indicated that the PIU is higher in the Unhappy group.

Similarly, the test assumptions for one-way ANOVA were checked to assess the differences in PIU across the different levels of participants' happiness with the frequency of phone checking. Levene's test was not significant (p-value = .466), indicating no violation of the homogeneity of variance. Normality was checked with a Q-Q plot, where no deviations were noted. There was a significant difference among the groups of happiness with the frequency of phone checking (F (3, 264) = 6.85, p < .001, η^2^ = .072). The post hoc test showed a significant difference only between the Unhappy group (M = 19.42, SD = 4.58) and Somewhat Unhappy group (M = 16.72, SD = 4.12) with p = <.001. The difference between the other groups was insignificant.

### 3.4 Objectively recorded usage, EI, and happiness with phone usage as predictors of PIU

A multiple regression analysis was conducted to examine the predictive effect of gender, age, amount of smartphone usage in its four types, EI components, happiness with phone usage, and happiness with the frequency of phone checking on PIU. Prior to performing the regression analysis, the assumptions were checked and verified. The dependent variable PIU was visually normally distributed, and in reference to Kim [70], skewness and kurtosis absolute z-value was <3.29. There were no outliers that significantly deviated from the model based on the standardized residuals, which did not exceed −3.29 and 3.29. The collinearity statistics confirmed no multicollinearity among the variables. VIF values were <5 (for all predictors, it was less than 2), and Tolerance values were >0.2. Pearson's correlation was also performed and showed no multicollinearity among the variables, as shown in Table 3. Durbin–Watson value was between 1 and 3, indicating the independence of predictors. The normality and homoskedasticity of the residuals were satisfied as the residual's histogram was roughly normally distributed. The Q-Q plot for residuals indicated most of the data points to be on or close to the line.

**Table 3:** Pearson's correlation between PIU, smartphone app types usage, trait EI components, happiness with phone usage and happiness with frequency of phone checking N (268).

Variable	1	2	3	4	5	6	7	8	9	10	11	12	13	14
1. PIU	—													
2. Obsession	0.797∗∗∗	—												
3. Neglect	0.749∗∗∗	0.399∗∗∗	—											
4. Control Disorder	0.779∗∗∗	0.415∗∗∗	0.390∗∗∗	—										
5. Avg comm. usage (min)	0.183∗∗	0.136∗	0.106	0.182∗∗	—									
6. Avg social media usage (min)	0.226∗∗∗	0.222∗∗∗	0.152∗	0.147∗	−0.008	—								
7. Avg gaming usage (min)	0.204∗∗∗	0.150∗	0.172∗∗	0.154∗	−0.008	0.098	—							
8. Avg other apps usage (min)	0.194∗∗	0.113	0.141∗	0.199∗∗	0.294∗∗∗	−0.004	0.081	—						
9. EI's Well-being	−0.287∗∗∗	−0.309∗∗∗	−0.153∗	−0.197∗∗	−0.022	−0.103	−0.192∗∗	−0.181∗∗	—					
10. EI's Self-control	−0.397∗∗∗	−0.346∗∗∗	−0.259∗∗∗	−0.313∗∗∗	−0.005	−0.124∗	−0.135∗	−0.048	0.566∗∗∗	—				
11. EI's Emotionality	−0.215∗∗∗	−0.147∗	−0.213∗∗∗	−0.145∗	0.104	−0.053	−0.011	−0.031	0.446∗∗∗	0.334∗∗∗	—			
12. EI's Sociability	−0.287∗∗∗	−0.170∗∗	−0.242∗∗∗	−0.261∗∗∗	−0.047	0.031	−0.156∗	−0.105	0.470∗∗∗	0.436∗∗∗	0.372∗∗∗	—		
13. Happiness with phone usage	−0.276∗∗∗	−0.206∗∗∗	−0.157∗	−0.276∗∗∗	0.022	−0.169∗∗	−0.033	−0.083	0.087	0.155∗	0.032	0.055	—	
14. Happiness with phone checking	−0.218∗∗∗	−0.125∗	−0.193∗∗	−0.193∗∗	−0.022	−0.021	−0.083	−0.048	0.079	0.117	0.020	0.102	0.606∗∗∗	—

∗p < .05, ∗∗p < .01, ∗∗∗p < .001.

As shown in Table 4, gender, age, the averages of each app types usage, the components of EI, happiness with phone usage, and happiness with the frequency of phone checking variables were included in the model as predictors and with PIU as the dependent variable. As it was observed in the data that there were participants with no social media usage or no gaming usage, two new dichotomous variables were created, “Has_Social_Usage?” and “Has_Gaming_Usage?”. These new variables differentiate those who used the respective category from those who did not and were added to the regression model as well. Overall, gender, and age were not statistically associated with PIU. The results also revealed that the average usage of communication apps, social media apps, and gaming apps and happiness with phone usage factors were significant and positive predictors of PIU. Trait EI self-control was a significant and negative predictor of PIU. The overall model contributed to the prediction of PIU with a variance of 32.5% (*R*^*2*^ = .325, adjusted *R*^*2*^ = .287 (*F* (14, 253) = 8.69, *p* < .001)). The strongest predictor was trait EI self-control (*ꞵ =* −.28, *p* < .001), followed by the average of social media usage (*ꞵ* = .19, *p* = .002), and then the average of communication usage (*ꞵ* = .17, *p* = .002) and happiness with phone usage (*ꞵ* = −.17, *p* = .013).

**Table 4:** Multiple regression analysis of smartphone app types usage, trait EI components, happiness with phone usage and happiness with frequency of phone checking predicting PIU N (268).

Predictors	Standardized *ꞵ*	t	*p*
**Constant**		12.85	<.001
Gender (Male/Female)	.01	0.13	.896
Age (15–24/25–64)	−.05	−0.84	.401
Avg comm. usage (min)	.17	3.10	.002
Avg social media usage (min)	.19	3.13	.002
Avg gaming usage (min)	.13	2.22	.027
Avg other apps usage (min)	.08	1.43	.154
Has_Social_Usage? (No/Yes)	−.10	−1.83	.069
Has_Gaming_Usage? (No/Yes)	−.01	−0.23	.817
EI's Well-being	.05	0.75	.452
EI's Self-control	−.28	−4.10	<.001
EI's Emotionality	−.10	−1.55	.123
EI's Sociability	−.12	−1.84	.067
Happiness with phone usage	−.17	−2.51	.013
Happiness with phone checking	−.06	−0.94	.350

R^2^= .325, R^2^Adj= .287, F (14, 253) = 8.69.

### 3.5 Objectively recorded usage, EI, and happiness with phone usage as predictors of PIU components

The assumptions of multiple regression were verified. The collinearity statistics confirmed no multicollinearity among variables as VIF values were <5 for all predictors and Tolerance values were >0.2. The Pearson's correlation also showed no multicollinearity among variables, as shown in Table 3. Durbin–Watson statistics value was between 1 and 3, confirming the independence of predictors. The normality and homoskedasticity of the residuals were satisfied as the residual's histogram showed roughly normal distribution. The Q-Q plot for residuals indicated most of the data points to be on or close to the line. There were no outliers that significantly deviated from the model based on the standardized residuals that did not exceed −3.29 and 3.29.

The multiple regression models, as shown in Table 5, included age, gender, the averages of each app types usage, EI's components, happiness with amount of time spent on phone, and happiness with frequency of checking the phone as predictors and each of the PIU components as the dependent variable. The variables contributed to the variance of obsession by 23.8% (*R*^*2*^ = .238, adjusted *R*^*2*^ of .196 (*F* (14, 253) = 5.64, *p* < .001)), the variance of neglect by 18.4% (*R*^*2*^ = .184, adjusted *R*^*2*^ of .139 (*F* (14, 253) = 4.07, *p* < .001)), and the variance of control disorder by 25.5% (*R*^*2*^ = .255, adjusted *R*^*2*^ of .214 (*F* (14, 253) = 6.18, *p* < .001)). In the three models, gender and age remained insignificant predictors of each PIU component. The average usage of communication apps, and social media apps, the dichotomous variable “Has_Social_Usage?”, EI's self-control, and happiness with phone usage contributed significantly to the prediction of PIU's obsession. The average usage of gaming apps, EI's self-control, and EI's emotionality were significant predictors of the PIU's neglect. The average usage of social media was close to significantly predicting PIU's neglect. The average usage of communication apps, EI's self-control, EI's sociability, and happiness with phone usage were significant predictors of PIU's control disorder. The strongest predictor of PIU's obsession was EI's self-control (*ꞵ =* −.23, *p* = .001) followed by the average social media apps usage (*ꞵ =* .20, *p* = .002). The strongest predictor of PIU's neglect was EI's self-control (*ꞵ= −.18*, *p* = .016) followed by EI's emotionality (*ꞵ= −.17*, *p* = .015). For PIU's control disorder, the strongest predictor was EI's self-control (*ꞵ= −.23*, *p* = .001) followed by happiness with phone usage (*ꞵ= −.21*, *p* = .003). In comparing the contribution of variables to the models of each PIU component, the variables were shown to contribute the highest to the variance of PIU's control disorder (25.5%).

**Table 5:** Multiple regression analysis of smartphone app types usage, trait EI components, happiness with phone usage and happiness with frequency of phone checking predicting PIU components (obsession, neglect, and control disorder) N (268).

Predictors	Obsession			Neglect			Control Disorder		
	Standardized ꞵ	t	p	Standardized ꞵ	t	p	Standardized ꞵ	t	p
Constant		8.97	<.001		8.66	<.001		10.32	<.001
Gender (Male/Female)	.03	0.40	.688	−.01	−0.22	.829	.00	0.06	.950
Age (15–24/25–64)	−.05	−0.87	.386	.03	0.43	.667	−.08	−1.33	.184
Avg comm. usage (min)	.15	2.46	.015	.09	1.44	.152	.16	2.81	.005
Avg social media usage (min)	.20	3.13	.002	.13	1.95	.052	.11	1.67	.096
Avg gaming usage (min)	.11	1.78	.076	.13	2.01	.046	.06	1.05	.297
Avg other apps usage (min)	.01	0.18	. 860	.11	1.65	.099	.08	1.37	.173
Has_Social_Usage? (No/Yes)	−.15	−2.44	.015	.01	0.17	.869	−.09	−1.56	.120
Has_Gaming_Usage? (No/Yes)	−.09	−1.50	.134	−.02	−0.26	.792	.08	1.35	.177
EI's Well-being	−.12	−1.58	.115	.15	1.90	.058	.11	1.53	.128
EI's Self-control	−.23	−3.23	.001	−.18	−2.42	.016	−.23	−3.23	.001
EI's Emotionality	−.01	−0.13	.900	−.17	−2.45	.015	−.06	−0.92	.360
EI's Sociability	−.00	−0.03	.974	−.13	−1.85	.066	−.15	−2.24	.026
Happiness with phone usage	−.16	−2.21	.028	−.01	−0.18	.855	−.21	−2.97	.003
Happiness with phone checking	.02	0.22	.828	−.14	−1.85	.065	−.04	−0.52	.607

Obsession: R^2^= .238, R^2^Adj= .196, F (14, 253) = 5.64.

Neglect: R^2^= .184, R^2^_Adj_= .139, F (14, 253) = 4.07.

Control Disorder: R^2^= .255, R^2^_Adj_= .214, F (14, 253) = 6.18.

## 4 Discussion

The current study adds to the literature by investigating the impact of smartphone apps usage based on objectively recorded data, EI Components, and subjective happiness with smartphone use factors on PIU and its components while taking participants’ demographic characteristics into account. The study is also one of the first to assess the risk factors for PIU in a diverse sample of participants from various age groups, living conditions, and countries.

From the multiple regression, it is apparent that the demographic characteristics of age and gender were not associated with PIU (RQ1). Our findings echo that of Beranuy et al. in [71], which showed that gender and age were not significantly associated with the PIU measured, in their study, through a Spanish scale CERI (Cuestionario de Experiencias Relacionadas con Internet). However, there is inconsistency in the literature studying the relationship between gender and PIU. For example, a finding in [72] revealed a higher PIU among females, whereas another study by Stavropoulos et al. [73] reported a higher PIU among males. Other studies concluded no gender-related differences in PIU [74, 75]. Similarly, there is an inconsistency in the results found in the literature regarding the association of age with PIU. In the study conducted by Ergin [76], the results indicated that PIU level increased positively with age. On the contrary, a positive association between PIU and being younger in age was found in [77, 78]. In addition, an insignificant relationship between PIU and age was seen in previous studies [79].

For the demographic characteristic of living conditions (RQ1), the one-way ANOVA analysis revealed that PIU was not significantly different among participants in different living conditions, whether they lived alone or with others. It could be argued that people living alone are more likely to feel loneliness, which was often found as a predictor for PIU [80]. The evidence in the literature on the relationship between living alone and loneliness showed mixed results [81]. In our study, the living condition was not a significant predictor of PIU, possibly because living alone does not necessarily indicate loneliness or perception of technology overuse as problematic.

The inconsistent findings on the association between gender, age, and living condition with PIU could be a result of the different age ranges of the participants or the scales used for PIU in the different studies. There are other environmental factors that could also impact this association. For example, PIU level was found higher in boys with higher internet access at home [76]. COVID-19 could be an environmental factor with which to interpret our results. During the period of our data collection (October 2020 to April 2021), restrictions on social gatherings were still applied. This means that people, regardless of their gender, age group, and living conditions, had a limited set of options to perform offline activities.

The multiple regression analysis showed that only subjective happiness with phone usage, communication usage, social media usage, gaming usage, and EI's self-control were found to be significant predictors of PIU (RQ2). Literature has shown that subjective positive feelings about own life conditions negatively correlate with PIU [82, 83]. However, research investigating the association between subjective feelings about smartphone use itself and PIU is lacking. It can be intriguing to find whether people's PIU, assessed by clinical and psychological measures, is associated with their subjective feelings about the use itself. In our study, the regression analysis revealed that subjective happiness regarding the amount of smartphone usage was negatively associated with PIU, suggesting that those being happy with the amount of smartphone usage tend to have lower PIU. The one-way ANOVA analysis further revealed that the PIU difference was more significant between each of the groups Happy, Somewhat Happy, and Somewhat Unhappy, on the one hand, and the Unhappy group, on the other. Although denial is a common characteristic of problematic internet behavior [84], the fact that our participants installed the monitoring app to be aware of their amount of usage can be an explanation for why being unhappy with phone usage is indeed associated with PIU. In other words, the instalment of our monitoring app, which allows the user to see how much they use their smartphone, indicates acceptance and receptiveness to objectivity in the participants towards their technology usage. For the happiness with the frequency of checking the phone, although it was not a significant predictor of PIU when combined with other factors in the regression model, the one-way ANOVA analysis revealed a significant difference between the Somewhat Unhappy and Unhappy groups. The frequent checking of the phone could be habitual and impulsive behavior which means it is an automatic process that occurs without attention or consciousness [85, 86]. For this reason, our measurement of people's feelings about their frequent checking of the phone could be inaccurate and therefore affected our findings of its association with PIU. Hence, future studies may use objective methods to measure the frequency of checking the phone and users' feelings about it.

The amount of communication usage, social media usage, and gaming usage were positively associated with PIU. The amount of social media usage had a higher impact on PIU than the usage of communication apps and gaming apps, which is consistent with the study in [37]. The increase in the amount of internet usage as a whole, whether the use is through smartphones or other devices, is expected during COVID-19 [6, 87]. A previous study revealed that the amount of social media usage increased during COVID-19 as it became a platform to retrieve information and share feelings [88]. Another study found that the amount of social media and communication apps usage increased during the COVID-19 lockdown and that the amount of gaming apps usage decreased during the first few weeks of the lockdown [89]. Hence, one may argue that when people find their increased amount of usage is justifiable by the lack of alternatives and the restrictions on social gatherings, they may not perceive themselves to have significantly higher PIU. However, our results still showed that the amounts of smartphone usage, in the three types of apps, were significant and positive predictors of PIU and its components even under conditions where overuse can be seen as the norm. The findings add to the growing body of evidence that spending more time on social media, communication, and gaming increases the risk of PIU. It may also contribute to the conceptualization of PIU by focusing on the types of internet usage.

In terms of emotional intelligence components, EI's self-control was negatively associated with PIU. EI could play a protective role against PIU [55] because people with higher EI can regulate their emotions and deal with life problems using adaptive strategies [90]. On the other hand, people with lower EI are more susceptible to developing problematic behaviors, including PIU as a strategy to cope with life problems. Nonetheless, there is still relatively limited research examining the relationship between EI components and PIU [52, 91]. Our findings supported previous studies that found correlations between EI and its components on the one hand and PIU on the other [52, 92, 93]. Although all EI components correlated negatively with PIU, only EI self-control contributed to predicting PIU in the regression model, suggesting that people with higher EI in term of controlling their emotions had lower PIU. The literature highlighted that impulse control is a key feature in behavioral addiction [94]. According to impulsiveness theory [95], individuals with high self-control (of behavior, thoughts, and emotions) are often less impulsive to engage in short-term rewarding behaviors such as PIU. This implies that the presence of protective factors, particularly self-control, may reduce the likelihood of having PIU. Moreover, EI's components of well-being, emotionality, and sociability did not significantly contribute to explaining PIU when combined with other variables in our regression model. Further research may extend the study and investigate the impact of EI components measured objectively on PIU.

For (RQ3), of the three regression models where the outcome were PIU components, the models' predictors contributed higher to the variance of the control disorder component. The regression model results also revealed that being happy with phone usage was a significant and negative predictor of the PIU's obsession and control disorder components. The amount of usage of communication apps was a significant and positive predictor of the PIU's obsession and control disorder components. The amount of usage of social media apps, and having social media apps, represented through the variable “Has_Social_Usage?” were significant and positive predictors of the PIU's component of obsession. The amount of usage of gaming apps was a significant and positive predictor of the PIU's neglect component. Our findings contradict a previous study that revealed no correlation between the amount of social media apps usage and gaming apps usage on the one hand and the PIU's obsession component on the other [96]. The findings of the previous study, unlike our study, was based on self-reporting measurement or usage. In our study, apps usage was calculated based on objectively recorded data. Our categorization of apps was retrieved from Google Play and then manually checked and corrected to ensure accuracy. It is worth noting that our results could be impacted by different factors, including that people may be playing games through other devices such as virtual reality devices and computers [97, 98] or through social media apps [99]. Hence, our measurement of technology usage considers smartphone usage only, which can be representative of communication apps usage and social media apps usage. It is not considered representative of gaming apps usage and, particularly, its overuse. Thus, our results can be a better fit for mobile gaming.

Additionally, there is scarce research exploring the association between trait EI components and the components—not only the total score—of PIU. Our study addressed this gap and showed significant and negative associations between trait EI components measured using TEIQue-SF and PIU measured using PIUQ–SF–6. This finding was in line with a previous study that investigated the association between trait EI components measured using TEIQue-SF and PIU using the Problematic Internet Use Questionnaire (PIUQ) scale [100]. The results of the regression models showed that EI's self-control component was a negative and significant predictor of the three PIU components, EI's emotionality was a significant and negative predictor of PIU's neglect, and EI's sociability was a significant and negative predictor of PIU's control disorder (RQ3). The findings suggest that different EI components may explain different symptoms of PIU, and further assessment of PIU may take into account examining the different components of EI.

The current study provided empirical evidence of the impact of different user's profile and personality factors on PIU and its components. The results can be utilized when developing educational or technological interventions for PIU. Educational programs may incorporate awareness of the overuse of the three types of apps investigated in our study as risk factors of PIU. They may also work on improving the EI skills of an individual as a strategy to prevent PIU, depending on the type of apps being overly used. Such educational programs about PIU risk factors and EI skills can be delivered through the same medium, e.g., technology, in a personalized way. According to research, personalized learning is an effective and motivating method for engaging learners and improving learning outcomes [101, 102]. Personalization adapts solutions based on the learner's needs and behaviors. For example, an individual can be taught about goal settings to limit their usage in a way related to their internet use behavior and EI skills. A study in [103] showed that goal setting and planning in a way adapted to an individual can enhance their EI skills and change their behavior. Given the empirical evidence from our findings that the EI's self-control component contributed the highest to the prediction of PIU and all its components, education programs may prioritize improving an individual's self-control of EI. Developers of technology may consider the role of EI and pay attention to EI's self-control component when developing PIU interventions assisted by the design of the technology itself. Such interventions may offer features that help users be aware of their EI and assist them to improve their EI skills, e.g., limit setting, reminders, and self-monitoring. The clinical implication of our research could be that the usage amount of the three app types and EI's self-control, may be prioritized when assessing PIU, while the amount of usage of communication apps and social media apps, as well as EI's self-control may be prioritized when assessing PIU's obsession. The usage amount of gaming apps and EI's components of emotionality and self-control may be prioritized when assessing PIU's neglect component. The usage amount of social media apps and EI's sociability and self-control components may be prioritized when assessing the control disorder component of the PIU.

The current study is subject to several limitations. Due to the cross-sectional design, our findings do not necessarily imply causal relationships among variables. Further longitudinal studies are needed to establish causal relationships. Although time spent on the internet and time spent on smartphones overlap [58], a study in [104] suggested distinguishing between both smartphone and non-smartphone internet usage. Our collected data were limited to internet usage via smartphone, and we assumed that smartphone usage is representative of technology usage for reasons unrelated to work or study. Still, there could exist some users who significantly use communication mobile apps, in particular, for work, especially since our data were collected at the time when Covid-19 restrictions on social gatherings were largely applied. There could also be users who use the internet through multiple devices, especially for playing games. Therefore, our results should be interpreted with caution that the usage we collected is not necessarily a reflection of the participant's overall technology usage, though using smartphone for social media and communication is mostly prominent. Furthermore, although the participants were from 10 countries, the majority were from Western countries (85.45%). The number of participants in the age group 45 years old and above was less in comparison to the other groups. The unbalanced groups in our sample make it difficult to generalize our findings. Among our participants were employees, and their amount of usage could have included usage for work purposes. Employed participants' amount of usage for work purposes was also more likely to increase during the period of our data collection due to the COVID-19 pandemic. The COVID-19 lockdowns and restrictions on social gatherings were still applied during our data collection period, and most of the employees were working from home [105]. Similarly, several educational institutions switched from classroom-based learning to online learning during COVID-19, leading students to spend more time on the internet [106]. Our student participants' usage for educational purposes may have increased during this time period. We have measured happiness with the time spent on smartphone and frequency of use through a 5-points Likert scale. A validated and more elaborated scale could have helped further results, e.g., happiness of use of each type of apps and at different times of the day. For example, previous research has shown a correlation between PIU and gaming use at night [40]. Our study was limited to investigating the impact of the amount of smartphone apps usage on PIU and did not consider the purpose of the usage due to the lack of such information in our data. Future work may further explore the impact of objective data of smartphone apps usage together with the purpose of usage on PIU and its components. The purpose of usage of smartphone apps could be measured objectively by utilizing the features of the apps. For example, when using the Facebook app, news feeds, posting, and reels features could be used to assess the purpose of usage, whether for reading news, sharing information, or watching videos.

## 5 Conclusion

PIU prediction allows for an early intervention to prevent it. PIU prediction models can utilize different personal metrics, including personality and emotional intelligence traits, and also behavioral metrics such as amount, frequency, and type of technology usage, and other life activities such as exercises. Our study investigated the impact of the different app types on PIU. This is one of the few to incorporate objective data about smartphone usage collected through a designated app to investigate the impact of the amount of usage on PIU. Data about the amount of usage is more likely to generate more reliable results when collected objectively. According to the findings, people with high amounts of communication apps usage, social media apps usage, and gaming apps usage are prone to have higher PIU. The amount of usage of other apps had no effect on PIU. The results provided empirical evidence to support the literature that the type of internet usage, rather than the overall time spent on the internet, is a better indicator of PIU [107]. Future work may further investigate the impact of apps usage categorized based on a fine-grained taxonomy on PIU. Future studies may also explore other measures of internet use, including the length of app usage sessions, the frequency of app launches, and the purpose of app usage. Our study also contributes to the growing literature of PIU by examining the association between trait EI components using TEIQue-SF and subjective level of happiness with phone usage, on the one hand, and PIU and its components measured using PIUQ–SF–6, on the other. The EI component of self-control has a higher impact on PIU than do other predictors in our regression models. The results showed that participants with a high ability to control their emotional difficulties are less likely to develop PIU. Literature has also demonstrated the role of self-control in developing problematic behaviors [108]. Further research can focus on whether self-control can be enhanced through designing software-based behavior regulations, e.g., goal and limit sitting [109]. Previous studies showed an increase in internet usage during COVID-19 [87]. Our data were collected during this period; further studies are also needed to understand whether the findings were influenced by the increased usage of the internet and the restrictions on social gatherings during this period. Several implications for our findings are identified. For example, clinical assessment of PIU may consider the self-control of a patient's EI and their usage amount of certain app types rather than the overall smartphone usage when diagnosing PIU and its components. Smartphone applications developed to assess PIU could be designed by considering the type of internet usage rather than smartphone usage as a whole. They may also integrate features that help improve the EI skills of an individual.

## Declarations

### Author contribution statement

Sameha Alshakhsi, Raian Ali: conceived and designed the experiments; contributed reagents, materials, analysis tools or data; performed the experiments; analyzed and interpreted the data; wrote the paper.

Khansa Chemnad, John McAlaney: performed the experiments; materials, analysis tools or data.

Mohamed Basel Almourad: conceived and designed the experiments; contributed reagents, materials, analysis tools or data.

Majid Altuwairiqi: contributed reagents, materials, analysis tools or data.

### Funding statement

This work was supported by Zayed University: R180053, Taif University: 1-441-79. Open Access funding was provided by the Qatar National Library.

### Data availability statement

Data will be made available on request.

### Declaration of interests statement

The authors declare no conflict of interest.

### Additional information

No additional information is available for this paper.
